# Bimodal action of the flavonoid quercetin on basophil function: an investigation of the putative biochemical targets

**DOI:** 10.1186/1476-7961-8-13

**Published:** 2010-09-17

**Authors:** Salvatore Chirumbolo, Marta Marzotto, Anita Conforti, Antonio Vella, Riccardo Ortolani, Paolo Bellavite

**Affiliations:** 1 Department of Pathology and Diagnostics, sect. General Pathology, strada Le Grazie 8, 37134 Verona, Italy; 2 Department of Medicine and Public Health-sect. Pharmacology, University of Verona, Italy, strada Le Grazie 8, 37134 Verona, Italy; 3 Immunopathology Service, University Hospital, Policlinico GB Rossi, piazzale AL Scuro 10, 37134 Verona, Italy

## Abstract

**Background:**

Flavonoids, a large group of polyphenolic metabolites derived from plants have received a great deal of attention over the last several decades for their properties in inflammation and allergy. Quercetin, the most abundant of plant flavonoids, exerts a modulatory action at nanomolar concentrations on human basophils. As this mechanism needs to be elucidated, in this study we focused the possible signal transduction pathways which may be affected by this compound. Methods: K2-EDTA derived leukocyte buffy coats enriched in basophil granulocytes were treated with different concentrations of quercetin and triggered with anti-IgE, fMLP, the calcium ionophore A23187 and the phorbol ester PMA in different experimental conditions. Basophils were captured in a flow cytometry analysis as CD123bright/HLADRnon expressing cells and fluorescence values of the activation markers CD63-FITC or CD203c-PE were used to produce dose response curves. The same population was assayed for histamine release.

**Results:**

Quercetin inhibited the expression of CD63 and CD203c and the histamine release in basophils activated with anti-IgE or with the ionophore: the IC50 in the anti-IgE model was higher than in the ionophore model and the effects were more pronounced for CD63 than for CD203c. Nanomolar concentrations of quercetin were able to prime both markers expression and histamine release in the fMLP activation model while no effect of quercetin was observed when basophils were activated with PMA. The specific phosphoinositide-3 kinase (PI3K) inhibitor wortmannin exhibited the same behavior of quercetin in anti-IgE and fMLP activation, thus suggesting a role for PI3K involvement in the priming mechanism.

**Conclusions:**

These results rule out a possible role of protein kinase C in the complex response of basophil to quercetin, while indirectly suggest PI3K as the major intracellular target of this compound also in human basophils.

## Background

Flavonoids include a large group of low molecular weight polyphenolic secondary plant metabolites which can be found in fruits and vegetables, and plant derived beverages such as tea, wine and coffee [1-3]. More recently, these natural compounds have been recognized to exert antioxidant [4], anti-bacterial and anti-viral activity, in addition to anti-allergic effects [5-7], and exert anti-inflammatory [8], anti-angiogenic, analgesic, cardiovascular-protective [9], anti-hypertensive [10], hepatoprotective [11], cytostatic, cancer preventive [12], apoptotic [13], estrogenic and even anti-estrogenic properties [14]. Quercetin (2-(3,4- dihydroxyphenyl)- 3,5,7- trihydroxy- 4H- chromen- 4-one) is the most abundant of the flavonoids and is commonly used as a food supplement [15], but evidence-based data regarding its clinical efficacy are quite scanty [16]. As well as many other flavonols, quercetin exerts many effects on inflammation and allergic responses. In this context quercetin is known mainly as a strong inhibitor of many effector functions of leukocytes and mast cells at the micromolar concentration range: the flavonoid is able to inhibit histamine release from human basophils activated with different agonists [17-19], to decrease the expression of the basophil activation markers tetraspan CD63 and ectoenzyme CD203c [20], to block mast cell degranulation in the rat cell line RBL-2H3 model [21], to inhibit the production of pro-inflammatory cytokines in HMC-1 mast cell line [22]. This evidence has led to the suggestion that quercetin might be a good candidate for immuno-modulation and anti-allergic therapy [23]. Moreover, recent evidence from our laboratory has reported that sub-micromolar concentrations of quercetin, while inhibiting basophil activation marker expression in cells stimulated through an IgE-dependent pathway, are able to prime those markers in a classical non IgE-dependent activation pattern, such as using a formylated peptide (fMLP) as soluble agonist [20]. The bimodal pattern showed by quercetin in basophils activated with fMLP, having the typical features of a classical hormetic mechanism [24,25], prompted further investigation.

Aiming at understanding the bimodal mechanism by which quercetin acts on human basophils, in this study we investigated some basic signaling events in basophil activation, such as calcium, protein kinase C (PKC) and PI3K. From a molecular point of view, quercetin has a significant bulk of intracellular targets, mainly serine/threonine and tyrosine kinases, which is very difficult to disentangle [26]. In basophil biology a first step to be forwarded could be investigating the differential pattern between anaphylactic degranulation and piecemeal degranulation, known to be related to IgE-mediated and to non-IgE mediated activation pathways, respectively and to the differential expression of surface molecules associated to cell activation [27]. This issue can be focused by the use of inhibitors and regulatory molecules able to dissect these mechanisms. We used a polychromatic flow cytometry approach [28] to investigate the effect of the flavonoid quercetin on the expression of membrane markers triggered by several different agonists in normal subjects (healthy screened blood donors). In parallel, we also evaluated whether the effects of quercetin on basophil membrane markers were reproduced using a classical assay of histamine release. The huge collection of quercetin effects on countless cellular kinases, transcription factors and regulatory proteins, claims for further investigation about the molecular nature of its pharmacological action. This study, in addition to representing a contribute to the comprehension of basophil biology, gives new clues about the modulatory role of this natural compound in cells of inflammation and allergy.

## Methods

### Materials

N-formyl-L-methionyl-L-leucyl-L-phenylalanine (fMLP), 4-(2-hydroxyethyl)-1-piperazineethanesulfonic acid (HEPES), quercetin dihydrate (minimum 98% HPLC), phorbol-12-myristate-13-acetate (PMA), the ionophore A23187, the PI3K inhibitor wortmannin, Na_3_- ethylendiaminetetraacetic acid (EDTA), sodium heparin, trypan blue and distilled water (HPLC grade, Chromasolv^® ^Plus) were all purchased from Sigma (Sigma-Aldrich GmbH, Germany). Goat anti-human IgE was purchased from Invitrogen-Caltag Laboratories, (UK). Histamine enzyme-linked immunosorbent assay (ELISA) releasing test was purchased from Labor Diagnostika Nord GmbH & Co., Germany. Mouse anti-human monoclonal antibodies for flow cytometry evaluation CD123-PECy5 (isotype IgG_1_, clone 6H6), CD45-APCCy7 (isotype IgG_1 _clone HI30), CD203c-PE (isotype IgG_1 _clone NP4D6), CD63-FITC (isotype IgG_1 _clone MEM-259) were purchased from Biolegend, San Diego CA, USA. HLA-DR-PECy7 (isotype IgG_2a _clone L243) was purchased from Becton Dickinson, Pharmigen CA, USA. Pure quercetin was dissolved in DMSO at a stock solution of 1 mg/ml and stored at +4°C for a maximum of 6 days; wortmannin was dissolved in DMSO at the stock concentration of 2 × 10^-3 ^M, stored at -20°C and thawed before use. Working solutions were made into HEPES modified buffer (20 mM HEPES, 127 mM NaCl, 5 mM KCl, 5 UI/ml sodium-heparin, pH 7.4) (HBE). fMLP was dissolved in dimethylsulfoxide (DMSO) as a 2 × 10^-2 ^M stock solution, stored at -20°C and thawed before use. The calcium ionophore A23187 and PMA were both dissolved in DMSO as stock solutions of 1 mg/ml (1.91 mM and 1.62 mM respectively), stored at -20°C and thawed before use. Working solutions of fMLP, anti-IgE, A23187 and PMA were freshly prepared in HBE supplemented with 5 mM CaCl_2 _and 2 mM MgCl_2 _(HBC buffer). All reagents were pure and quality checked; whenever necessary disposable plastic ware and sterile apyrogenic solutions were used.

### Subjects and sampling

A total of 70 blood donors volunteers (47% male, 53% female) were enrolled in this study. Recruitment was randomized and encompassed an age range from 24 to 65 yrs (mean 44.61 ± 4.57 SD) in order to have a wide experimental population and to prevent age influence on cell releasability [29]. All the subjects recruited in the study were non allergic and non atopic, they did not suffer of any immunological disorder and had never reported any previous history or genetic diathesis of chronic allergy; moreover, none underwent neither drug therapy nor anti-histamine therapy during the 48 hrs before the peripheral venous blood withdrawal. All participants completed and signed a specific consenting form for taking the samples and for data processing.

### Cell recovery and preparation

Basophils were collected as leukocyte-enriched buffy coats from venous K_2_-EDTA anticoagulated peripheral blood from four screened healthy donors in each experiment performed, according to previously described methods [28]. Buffy coats were pooled and suspended in HBE buffer. To count basophils and evaluate yields, an aliquot of about 1 ml of the cell culture was transferred to a Bayer ADVIA 2120 automated hematocytometer [30]. The volume of working cell suspensions was adjusted with HBE buffer in order to get a basophil count of 90-150 basophils/μl. Compared with hemocytometer counts of starting whole blood (30-50 basophils/μl), an average enrichment of about 1.5-3.0 times (mean = 2.4) was currently obtained. Trypan blue exclusion test revealed that 98.7% ± 7.4 SD leukocytes were viable. Aliquots (100 μl) of cell samples were incubated at 37°C for 10 minutes with an equal volume of HBE in the absence or in the presence of quercetin or wortmannin at the indicated final doses. Activation was performed by adding 50 μl of treated cells to 50 μl of HBC buffer containing 200 nM fMLP or 8 μg/ml of goat anti-human IgE or 1.0 μM A23187 or 100 nM PMA, according to the different protocols. Resting assays were performed by incubating cells in HBC buffer without agonists. Incubation was carried out at 37°C for 30 minutes and blocked by adding 100 μl of ice-cold HBE supplemented with 2.8 mM sodium-EDTA (Na_3_-EDTA). Then the samples were put on ice and stained with monoclonal antibodies (20 minutes at +4°C), according to previously published methods [28]. Afterwards, red blood cells underwent lysis with an ammonium-buffered solution (155 mM NH_4_Cl, 10 mM Na_2_HCO_3_, 0.10 mM Na_3_EDTA, pH = 7.2) for 4 minutes at +4°C; then samples were centrifuged at 700 *g *and pellets recovered and re-suspended in a PBS-buffered saline solution (pH 7.4) for flow cytometry reading.

### Histamine release

Cells treated with different concentration of quercetin and activated with the indicated agonists, were pelleted at 6000 rpm for 5 minutes and surnatants collected for a competitive histamine ELISA test. 25 μl of each sample was treated with buffers and an acylation reagent, incubated for 1 hour at r.t. and diluted with 200 μl of distilled water. Aliquots of 20 μl of these acylated samples were incubated overnight with 100 μl of antiserum, washed 3 times, incubated for 1 hr at r.t. with a horseradish peroxidase-conjugate, washed 3 times with washing buffer, incubated for 30 min at r.t. with the colorimetric substrate tetramethylbenzidine and reaction stopped. The absorbance of the solution in each wells was read within 10 minutes at 450 nm with a reference wavelength of 620 nm. Histamine was calculated as ng/ml of the released amine against the corresponding standard concentrations in the calibration curve.

### Flow cytometry and data processing

Basophil membrane markers were evaluated by flow cytometry using a five-color fluorochrome panel including CD45-APCCy7, CD123-PECy5 and HLA-DR-PECy7 as phenotyping markers and CD63-FITC and CD203c-PE as activation ones [28]. Flow analysis was performed using a 488 nm-633 nm two-laser BD FACScanto flow cytometer: the instrument had a 10,000 events/sec capability, six-color detection and 0.1% sample carryover. Analysis were performed with a mean flow rate of 300-500 events/sec, setting an excess limit of 50,000 events to record in the basophil gate in order to analyze the whole buffered suspension volume and having a proper estimation of cell recovery and reproducibility. Compensation followed cytometer manufacturer's instruction according an off-line procedure by applying automated electronics algorithms and preset templates, by using biparametric logarithmic dot plots, gate-specific tubes and single-tube data analysis, and optimizing FSC threshold and fluorochrome voltage as set up parameters. Mean of fluorescence intensity (MFI) was calculated automatically by the cytometer software. Percentage of activated cells was calculated by the software considering the CD63 expressing cells (CD63-FITC^positive ^cells) counted to the right of a threshold that was established including the main peak of fluorescence of a sample of resting cells. In order to reduce standard deviation due to positive fluorescent cells respect to negative or dimly ones, a logarithmic scale and a coefficient of variation to measure variability dispersion were used.

### Statistics

Data were analyzed using the software SPSS, version 11 for Windows, Chicago, IL. Dose response curves were obtained by plotting the triplicate data and their mean values and S.E.M. for each experiment using the Sigma plot 10 software. Kolmogorov-Smirnov and Shapiro-Wilk goodness-of-fit tests were performed to determine whether the sample population followed a Gaussian distribution. Differences between quercetin-treated and non-treated cells were analyzed by using a one-way analysis of variance (ANOVA) followed by Fisher LSD test. A value of *p *< 0.05 was considered statistically significant. IC_50 _was calculated for each curve of percentage of effect/control by a linear regression calculation according to the four parameter logistic model (4PL), also called the Hill-Slope model.

## Results

### Basophils stimulated with anti-IgE or fMLP

**Figure 1:**
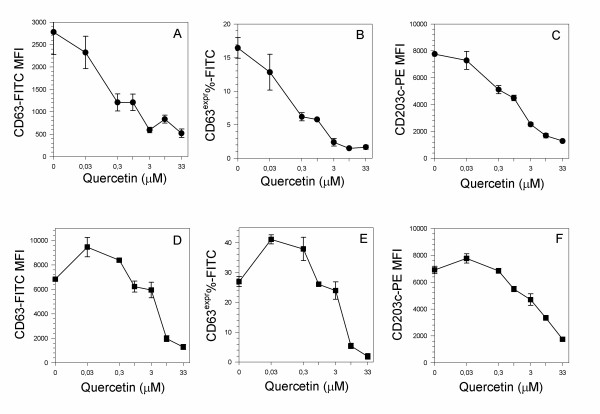
**Dose response of quercetin on membrane markers expression by anti-IgE (A,B,C) and fMLP (D,E,F) activated basophils**. Cells were pre-treated for 10 min at 37°C with increasing doses of quercetin, then stimulated for further 30 min at 37°C with 4 μg/ml anti-IgE or 100 nM fMLP, then evaluated as CD63 MFI (A, D), as the percentage of cells expressing CD63 marker (B, E) and as CD203c MFI (C, F).Values are mean ± S.E.M. of triplicate assays. Figure is representative of one triplicate experiment of 4 performed.

Figure 1 shows the dose response of the flavonoid quercetin on the expression of human basophil activation markers CD63 and CD203c following stimulation with 4 μg/ml anti-IgE (panels A,B,C) or 100 nM fMLP (panel D,E,F). In basophils stimulated with anti-human IgE, quercetin was able to inhibit in a dose-dependent fashion both the tetraspan (Figure 1, panels A,B) and the ectoenzyme up-regulation (Figure 1 panel C); quercetin IC_50 _able to inhibit CD63 expression was about 0.132 μM (values were 0.119 μM and 0.136 μM for CD63-MFI and for CD63^expr%^, respectively) while IC_50 _was about 6-fold higher (0.775 μM) for CD203c. In basophils activated with formylated peptides the flavonoid inhibited the activation markers at concentrations higher than 1.0 μM while it enhanced the same response at the doses of 0.033 μM and 0.33 μM (Figure 1 panels D,E), showing a typical bimodal hormetic pattern. Priming phenomenon (from 138.5% to 152.4% of untreated cells) was observed with the lowest quercetin concentration used in the assay, namely 0.033 μM and appeared more pronounced for CD63 expression than for CD203c one (Figure 1 panel F).

### Basophils stimulated with the calcium ionophore A23187 or with PMA

**Figure 2:**
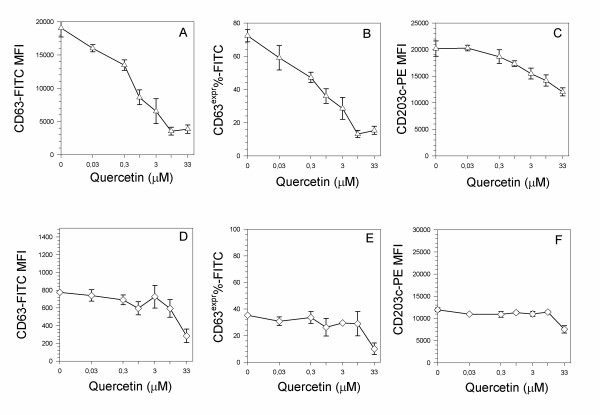
**Dose response of quercetin on membrane markers expression by calcium ionophore (A,B,C) and PMA (D,E,F) activated basophils**. Cells were pre-treated for 10 min at 37°C with increasing doses of quercetin, then stimulated for further 30 min at 37°C with 0.5 μM calcium ionophore A23187 or 50 nM PMA, then evaluated as CD63 MFI (A, D), as the percentage of cells expressing CD63 marker (B, E) and as CD203c MFI (C, F). Values are mean ± S.E.M. of triplicate assays. Figure is representative of one triplicate experiment of 4 performed.

Quercetin showed also a marked ability to decrease in a dose-dependent fashion the expression of CD63 in basophils activated with the calcium ionophore A23187 (Figure 2, panels A,B). Values of IC_50 _for CD63-MFI and for CD63^expr^% were respectively 0.573 μM and 0.824 μM. The expression of CD203c was much more resistant to the inhibition by quercetin (Figure 2, panel C): this evidence suggests some dissociation concerning the action of quercetin on calcium signaling of the two activation markers.

**Figure 3:**
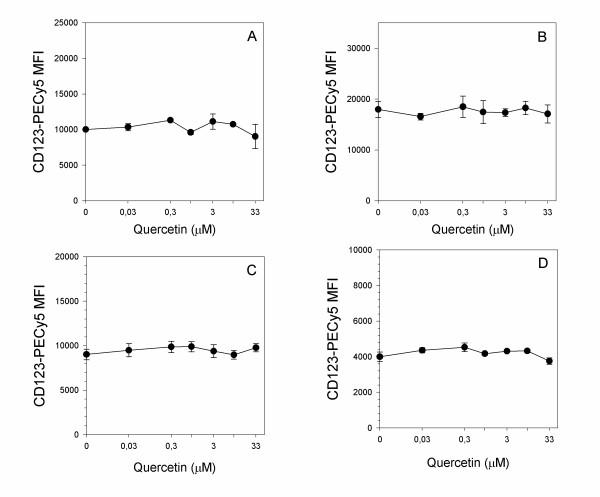
**Dose response of quercetin on membrane markers expression, evaluated as CD123-PECy5 MFI, in basophils activated with 4 μg/ml anti-IgE (A), 100 nM fMLP (B), 0.5 μM calcium ionophore A23187 (C) or 50 nM PMA (D)**. Cells were pre-treated for 10 min at 37°C with increasing doses of quercetin, then stimulated for further 30 min at 37°C with the indicated agonists. Values are mean ± S.E.M. of triplicate assays. Figure is representative of one triplicate experiment of 4 performed for each series of different stimuli.

When basophils, following pre-incubation with different concentrations of quercetin, were stimulated with the PKC activator PMA (Figure 2, panels D,E,F), the flavonoid did not show any significant inhibitory effect, except for the highest concentration used in the experiments (33 μM). The effect of quercetin showed a specificity for the activation markers, as other molecules used to phenotype basophils, such as CD123, which recognizes the alpha subunit of the constitutive basophil IL-3 receptor, was not affected by any of the quercetin concentrations used in any of the activation model considered in the study (Figure 3).

### Basophil releasability by the histamine ELISA test

**Figure 4:**
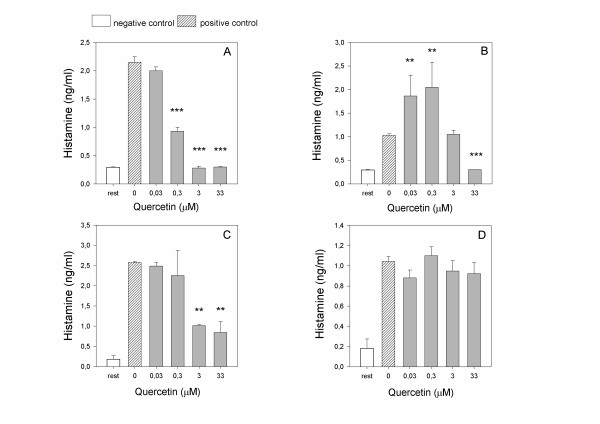
**Quercetin dose-response evaluated by assaying histamine release with an ELISA commercial kit**. Histamine is expressed as ng/ml of the vasoactive amine. Basophils were pre-treated 10 min at 37°C with increasing doses of quercetin and subsequently stimulated for further 30 min at 37°C with 4 μg/ml anti-IgE (A), 100 nM fMLP (B), 0.5 μM calcium ionophore A23187 (C) or 50 nM PMA (D). Values are mean ± S.E.M. of 3 different triplicate assays. Negative control: cells which did not undergo any treatment with quercetin and the agonist (resting cells); positive control: cells treated with the agonist but not treated with quercetin. One way global ANOVA for activated samples series: A) p < 0.0001; B) p < 0.001; C) p = 0.034; D) p = 0.65. Post-hoc (LSD) analysis of each dose as compared to positive control is indicated above the respective bar chart as: (**) p < 0.01; (***) p < 0.001.

Basophils treated with increasing doses of pure aglycone-quercetin were triggered with the different agonists used in the study and the histamine released after 30 minutes of incubation at 37°C was assayed with a competitive ELISA kit. Results are described in Figure 4: basophil releasability (degranulation) exhibited the same dose response behavior performed by the activation marker, particularly for CD63, namely a strong dose-response inhibition following anti-IgE activation (Figure 4, panel A), a bimodal pattern in the bacterial peptide activation protocol (Figure 4, panel B), a dose-response inhibition in the calcium ionophore stimulatory assay (Figure 4, panel C), and no inhibitory effect in the PMA activation pattern (Figure 4, panel C). The same cell population was investigated in the same experimental setting about CD63 and CD203c and CD123 membrane expression by flow cytometry: the behavior of these markers under the effect of quercetin was similar, in the various experimental conditions, to the behavior of histamine (data non shown).

### Wortmannin dose-response

**Figure 5:**
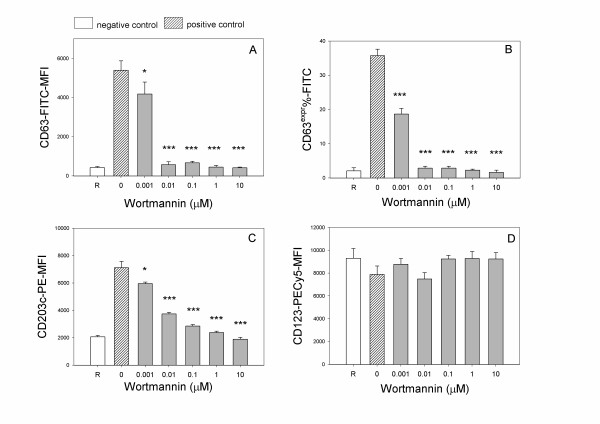
**Wortmannin dose-response in anti-IgE activated basophils, by assaying CD63 MFI (A), CD63^expr ^% (B) CD203c MFI (C) and CD123 MFI (D)**. Basophils were pre-treated 10 min at 37°C with increasing doses of wortmannin and subsequently stimulated for further 30 min at 37°C with 4 μg/ml anti-IgE. Values are mean ± S.E.M. of triplicate assays of 2 separate experiments performed. Negative and positive controls as in legend of Figure 4. One way global ANOVA for activated samples: A) p < 0.0001; B) p < 0.0001; C) p < 0.001; D) p = 0.76. Post-hoc (LSD) analysis of each dose as compared to positive control is indicated above the respective bar chart as: (*) p < 0.05; (***) p < 0.001.

**Figure 6:**
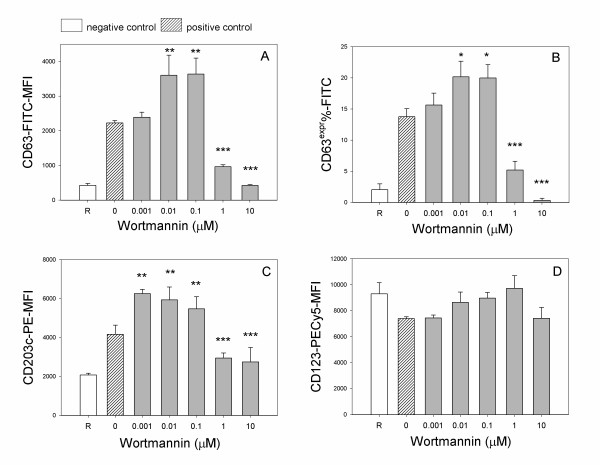
**Wortmannin dose-response in fMLP-activated basophils, by assaying CD63 MFI (A), CD63^expr ^% (B) CD203c MFI (C) and CD123 MFI (D)**. Basophils were pre-treated 10 min at 37°C with increasing doses of wortmannin and subsequently stimulated for further 30 min at 37°C with 100 nM fMLP. Values are mean ± S.E.M. of triplicate assays of 2 separate experiments performed. Negative and positive controls as in legend of Figure 4. One way global ANOVA for activated samples: A) p < 0.001; B) p < 0.001; C) p < 0.001; D) p = 0.69. Post-hoc (LSD) analysis of each dose as compared to quercetin untreated cells is indicated above the respective data point as: (*) p < 0.05; (**) p < 0.01; (***) p < 0.001.

Taking into account the two main activation protocols, namely the IgE-mediated and the fMLP mediated stimulation, basophils were treated with the specific PI3K inhibitor wortmannin in order to focus on a possible pathway involved in the bimodal behavior observed with the different agonists: the overall impression is that wortmannin behaved similarly to quercetin in our tested models. Figure 5 shows the dose response of wortmannin on basophils triggered with 4 μg/ml anti-IgE: it showed a pronounced inhibitory activity, with an IC_50 _of 2.17 × 10^-9 ^M and 1.99 × 10^-9 ^M for CD63 (Figure 5 panels A,B respectively) and 2.63 × 10^-9 ^M for CD203c (Figure 5, panel C). When basophils were activated with a formylated peptide, wortmannin showed a strong inhibitory action in the micromolar range and an increasing expression of CD63-MFI and of CD203c in the nanomolar range (Figure 6 panels A,B,C), surprisingly performing a biphasic or hormetic behavior as like as quercetin. Wortmannin, too, did not affect significantly the expression of a non activable marker such as CD123 (Figures 5 and 6, panel D).

## Discussion

**Figure 7:**
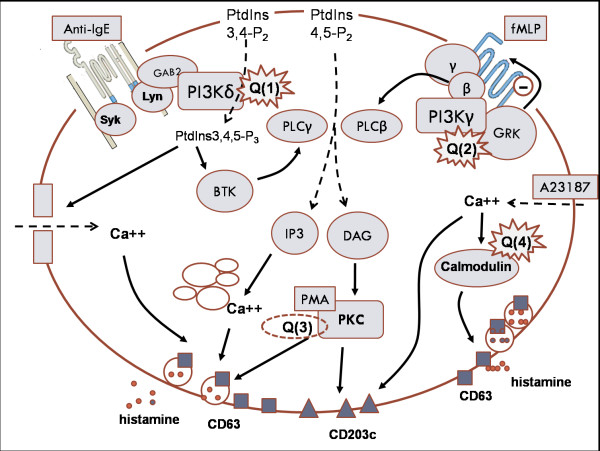
**Cartoon describing the possible pathways of inhibition or of priming/activation by quercetin (Q) in the activation models explored in the study**. Putative sites sensitive to quercetin are indicated close to the target proteins PI3Ks or calmodulin: for PKC, Q is included in a dashed area, indicating no effect of the flavonoid on the kinase. Membrane markers are indicated by squares (CD63) or triangles (CD203c). Arrows indicate links between different activation pathways while dashed arrows indicate precursors or metabolites incoming to the interior of the cell from membrane and/or from the outside. For further explanation and comments see the text. BTK: Bruton's tyrosine kinase; DAG: diacylglycerol; GAB-2: Grb-associated binding protein-2, an adaptor protein serving as principal activator of PI3K; GRK: G-coupled receptor kinase; IP3: inositol-1,4,5-triphosphate; PLC: phospholypase C; PtdIns 3,4-P_2_: phosphatidylinositol 3,4-biphosphate; PtdIns 4,5-P_2_: phosphatidylinositol 4,5-biphosphate; PtdIns 3,4,5-P_3_: phosphatidylinositol 3,4,5-triphosphate; Syk, Lyn: signaling tyrosine kinases linked to IgE-high affinity receptor; other abbreviations as in the text.

The results here presented confirm the inhibitory action of relatively high concentrations of quercetin on human basophils function previously reported by others [19,31,32] and by us [20] and assess putative mechanisms of the observed effects at the nanomolar dose range. Quercetin has many targets among intracellular kinases involved in many steps of receptor downstream signaling, leading to various effector functions, such as the degranulatory event [26] but its strong inhibitory action has usually been shown at the high micromolar concentration range, where biphasic effects were not reported. At highest micromolar doses, quercetin actually inhibits a variety of intracellular kinases but at a concentration range from 10^-7 ^M to 10^-8 ^M the action of quercetin might depend on more specific and sensitive steps of the activatory pathway used by the cell, probably on the receptor signaling complex. The importance of distinguishing the effects on the basis of *in vitro *acting dose range is also related to the evidence reported elsewhere by *in vivo *studies that the plasma concentration of quercetin in healthy volunteers following food supplementation ranged from 0.43 μM to 1.5 μM [33-35]. Here below, we would discuss the ability of quercetin to act as a modulatory compound in a sub-micromolar/nanomolar concentration range, taking into account Figure 7 as the summarizing picture of our hypotheses.

Q1) Effects of quercetin in the FcεRI-anti-IgE activation model. Previously published reports have shown that quercetin is able to inhibit PI3K by binding to the catalytic pocket of the enzyme: as for instance, LY294002, a synthetic inhibitor of PI3K, has actually a chemical kinship with the flavonoid quercetin [36]. The IC_50 _for quercetin as an inhibitor of PI3K from human blood platelets is around 1.8-20 μM [37], which corresponds to the inhibitory range observed in the results here reported on basophils. Taking into account the downstream signaling pathway of FcεRI-anti-IgE complex, a suggestion would come that the inhibition of PI3K leads to the loss of phosphorylation of downstream kinases such as Bruton's tyrosine kinase (BTK) [38] which in turn is able to phosphorylate PLCγ, thus leading to the production of inositol-1,4,5-triphosphate (IP_3_) and to diacylglycerol (DAG) from the precursor phosphatidylinosytol-4,5-biphosphate (PtdIns 4,5-P_2 _or PIP_2_) (Figure 7). While DAG remains to the membrane, IP_3 _diffuses to the cytosol and binds to and activates the InsP_3 _receptor on the membrane of the endoplasmic reticulum, opening a calcium channel, resulting in the release of Ca^2+ ^into the cytoplasm. DAG is able to activate PKC, which in turn activates membrane markers up-regulation and histamine release [39]. Warner et al. observed that the amount of histamine release associated with activation of basophils through IgE receptor aggregation, among different preparations of basophils, was correlated with an increase in membrane bound PKC-like activity [40]. These results also suggested that PKC activation may have a role in IgE-mediated histamine release in human basophils and that quercetin might inhibit basophil function by blocking DAG precursor for PKC in the upstream signaling pathways. The inhibition of PI3K by quercetin would also prevent the formation of phosphatidylinositol 3,4,5-triphosphate (PtdIns3,4,5-P_3_) which activates extracellular calcium influx by membrane Ca^++ ^channels [41].

Q2) Effects of quercetin in the FPR-fMLP activation model. Figure 7 depicts a speculative suggestion concerning the priming phenomenon observed on the fMLP-triggered basophil function. Since in our assay system the effects of quercetin were superimposable to those of wortmannin, a potent PI3K inhibitor [42,36], our results indirectly suggest a role for PI3K in the dual effects performed by the flavonoid. G-protein coupled receptors, such as the fMLP receptor, activate the PI3Kγ isoform through interactions with Gβγ of the PI3K p101 and p110γ subunits [43]. Increasing evidence suggests that monomeric p110γ may function as a downstream regulator of G-protein coupled receptor dependent signal transduction [43]: Gβγ is able to activate a G-coupled receptor kinase (GRK) which desentitizes the receptor. Interactions of quercetin with this Gβγ-p101/p110γ might exert an action leading to these possible results: a) the inability of Gβγ sequestered by p101/p110γ complex to activate G-coupled receptors kinases (GRKs) and to desensitize the receptor, leading to a priming mechanism for example by inducing a sustained activation of downstream protein kinases involved in the degranulatory event, such as p38-MAPK [44]; b) the long-lasting activation of Gβγ-associated PLCβ due to a defect in the Gβγ/PI3K dissociation, leading to an increase in signaling mediators able to trigger the degranulation event (by IP_3_-calcium signaling or by the activation of DAG-PKC pathway), so resulting in a priming effect.

Q3) Effect of quercetin on protein kinase C (PKC) activation pathway. In our assay system the flavonol proved insensitive to target protein kinase C (PKC), as resulted from the use of PMA as basophil stimulant, thus confirming previous reports [19]: quercetin was unable to inhibit CD63 and CD203c membrane up-regulation in basophils stimulated with phorbol esters and no dissociation between the two markers investigated was actually observed by using PMA [45]. So, the PKC pathway triggered by PMA, and presumably by other physiologic stimulants, is a quercetin-insensitive route to basophil activation.

Q4) Effect of quercetin on basophils triggered with calcium ionophore A23187. In response to calcium ionophore A23187 the expression of both the activation markers CD63 and CD203c was markedly up-regulated, but quercetin exerted a significant inhibitory action, even at nanomolar doses, only on CD63, so dissecting the response of the two activation markers to the ionophore. Previous evidence has reported that CD203c and CD63 upregulation in response to calcium signal by A23187 showed different kinetics [28], an evidence that probably suggests different pathways of calcium involvement in the expression of the two markers [46]. Our results indicate that the calcium-mediated signaling is essential both for the LAMP-1 CD63 and for the ENPP-3 CD203c upregulation, as A23187-mediated calcium influx stimulate both the expression of basophil activation markers and histamine release (see Figure 7), but on the same time they suggest also that the transduction pathway diverges in two distal branches, one of which (LAMP-1) is sensitive to quercetin and is related to the degranulatory event [47], the other is much more resistant to this inhibition. It is well known that A23187 promotes the activation of Ca++/calmodulin pathway [48], which is inhibited by quercetin [49]. Calmodulin constitutes an obligate link in signal transduction pathways leading to human leukocyte histamine release if the trigger is a calcium ionophore but not when responses are induced by anti-IgE, fMLP or PMA [48]. Quercetin ability to target calmodulin drives to the suggestion that those events inhibited by the flavonoid, i.e. the histamine release and CD63 membrane up-regulation, were presumably related to a Ca++/calmodulin dependent pathway in basophils activated with A23187, while the expression of CD203c, which was not significantly affected by the flavonoid even at its highest dose, might be a calmodulin-independent event. This marker is probably translocated to the membrane by other calcium dependent vesicular-transport mechanisms [50].

These hypotheses and models need for further investigation on a molecular level such as a direct demonstration of the kinases isolated from or detected in the purified basophils and/or by using isoform-selective inhibitors of PI3K and to assay calmodulin involvement in the A23187 activation pathway inhibited by quercetin. What is really interesting is that the observed modulatory biphasic (hormetic) mechanism can be related to the inhibition of PI3K by quercetin and that the efficacious doses are within the nanomolar plasma concentrations reported in several pharmacokinetic and bioavailability studies about this flavonoid [51-53]. At these concentrations is commendable that quercetin exerts a fine regulatory action depending on the fine balancing of signaling proteins ruled by PI3Ks. The PI3Ks seem to be strategic both for the activation of downstream protein kinases and for receptor-associate phospholypases C activation thus leading to calcium elevation in the cytoplasm and to PKC-mediated degranulation, two conditions which basophil needs for up-loading its markers of activation on the membrane and for histamine release. This might be a first step by which quercetin is able to exert its action at sub-micromolar-nanomolar concentration range, while at the highest doses its action might involve also other receptor and PI3K-downstream kinases such as Akt/PKB, MEK, p38-MAPK, etc. [26].

Allergy is a cause for concern, mainly due to its rising prevalence inside the population and to the increasing difficulty in treating chronic allergy. Quercetin might be a good candidate with the potential to counter this trend: an appropriate intake of this flavonol from food and beverage or from supplemental administration could be expected to improve allergy, to help anti-inflammatory and anti-oxidative responses by the organism and to prevent the onset of allergic chronic diseases. However our results introduce a caveat: although basophils play an important role in mediating allergic response and quercetin has proved to have an inhibitory action on basophils following stimulation with anti-IgE and calcium ionophore A23187, the existing bimodal effects of the flavonol and the complex nature of hypersensitivity reactions would require researchers be more cautious before considering quercetin in the practical use of the therapy and prevention of allergy. To achieve this goal, further research insights about cell signaling and about quercetin intracellular targets and studies in animal models are required.

## List of abbreviations

DMSO: dimethylsulfoxide; EDTA: ethylendiaminetetraacetic acid; ELISA = Enzyme-linked immunosorbent assay; ENPP-3: ectonucleotide pyrophosphatase phosphodiesterase-3. DAG: diacylglicerol; fMLP: N-formyl-L-methionyl-L-leucyl-L-phenylalanine; GRK: G-coupled receptor kinase; HEPES: 4-(2-hydroxyethyl)-1-piperazineethanesulfonic acid; HMC-1: human mast cell line-1; LAMP-3: lysosome associated membrane protein-3; MFI: Mean of fluorescence intensity; PI3K: phosphoinositide-3 kinase; PKC: protein kinase C; PLC: phospholypase C; PMA: phorbol-12-myristate-13-acetate; p38MAPK: p38-mitogen activated protein kinase; PtdIns3,4,5-P_3_: phosphatidylinositol-3,4,5-triphosphate; RBL-2H3: rat basophilic leukemia cell line.

## Competing interests

The authors declare that they have no competing interests.

## Authors' contributions

All the authors read and approved the final manuscript. SC designed the research, conducted in vitro analysis, discussed the results and wrote the manuscript. MM conducted some in vitro analysis and discussed the manuscript; AC discussed the pharmacological aspects of the manuscript; RO and AV managed the cytofluorimetric analysis; PB directed the research and revised the manuscript.
